# High Accuracy Localization of Long Term Evolution Based on a New Multiple Carrier Noise Model

**DOI:** 10.3390/s141222613

**Published:** 2014-11-27

**Authors:** Wah Ching Lee, Faan Hei Hung, Kim Fung Tsang, Chung Kit Wu, Hao Ran Chi, Kwok Tai Chui, Wing Hong Lau

**Affiliations:** 1 Department of Electronic and Information Engineering, Hong Kong Polytechnic University, Hong Kong; E-Mail: enwclee@polyu.edu.hk; 2 Department of Electronic Engineering, City University of Hong Kong, Hong Kong; E-Mails: fhhung4@cityu.edu.hk (F.H.H.); chungkwu4@cityu.edu.hk (C.K.W.); haoranchi2-c@my.cityu.edu.hk (H.R.C.); ktchui3-c@my.cityu.edu.hk (K.T.C.); itwhlau@cityu.edu.hk (W.H.L.)

**Keywords:** outdoor localization model, multiple carrier noise, LTE

## Abstract

A high accuracy localization technique using Long Term Evolution (LTE) based on a new and accurate multiple carrier noise model has been developed. In the noise consideration, the LTE multiple carriers phase noise has been incorporated so that a new and accurate noise model is achieved. An experiment was performed to characterize the phase noise of carriers at 2 GHz. The developed noise model was incorporated into LTE localization analysis in a high traffic area in Hong Kong to evaluate the accuracy of localization. The evaluation and analysis reveals that the new localization method achieves an improvement of about 10% accuracy comparing to existing widely adopted schemes.

## 1. Introduction

One of the prominent methods for localization employs wireless sensor nodes and directional antenna in robots to assist positioning [1,2] and results are encouraging. Since there are increasing numbers of Long Term Evolution (LTE) cellular phone subscribers, in this letter, LTE localization is investigated in an attempt to expedite consumer mobile applications. LTE has become the main-stream in mobile communication since it offers efficient spectrum utilization, cell coverage and handover compared with evolved high speed packet access (HSPA+) [3]. Typical localization algorithms employ received signal strength (RSS) to locate the mobile handset (MH) [4]. Since noise is the key factor influencing the accuracy of localization, a new and accurate noise model should be developed and applied to localization to enhance the localization accuracy.

The phase noise is an inherent property of a carrier that affects transmission performance. Since LTE is a multiple carrier scheme thus the phase noise incurred may affect the accuracy of localization. It is intuitive that the accuracy of localization should be improved if a more accurate noise model is developed. This investigation is dedicated to a new model that incorporates phase noise for multiple carriers. In telecommunication, RSS is an indicative parameter reflecting the strength of the received signal [5–8]. Normally, when the distance between a MH and the base station (BS) is short, the RSS will be large. In general, RSS can be greatly attenuated by tall buildings in dense or populated areas, hence deteriorating the accuracy of localization.

In this Communication, a new noise model dedicated to LTE localization of mobile handsets is proposed. Based on the developed noise model, a new localization model has been developed and an improvement of localization accuracy has been achieved. In mobile telecommunication, broadband Gaussian noise prevails in all circumstances and is incorporated into localization considerations [9]. In general, the Gaussian noise model [10] is adopted. In essence, the phase noise is an inherent property of the signal carrier. Hence the phase noise is an indispensable element in the noise evaluation. The multiple carrier nature of LTE further demands the consideration of the impact of the phase noise. In this investigation, it is proposed that the Gaussian noise co-exists with the phase noise in an LTE orthogonal frequency division multiplexing (OFDM) system.

## 2. Noise Model

For radio frequency propagation, a path-loss model [4,9] is typically used to determine the distance from BS to MH. The model considers the coordinates of BS and MH as well as the log-normal shadowing [11,12] which is represented by w*_o_*. *P_t_* is the transmitted power from BS and *P_r_* is the received power in MH, *P_L_* accounts for the reference signal power in one meter. *d_k_* is the distance between a BS and a MH and is associated with random Gaussian variable *w_o_*. Equation (1) is normally used to locate a handset's coordinates:

(1) $$10\log_{10}\;\left(\frac{P_{t}}{P_{r}}\right)\;\left(\text{dBm}\right)=P_{L}+10\alpha \log_{10}d_{k}+w_{o}$$

**Figure 1.**
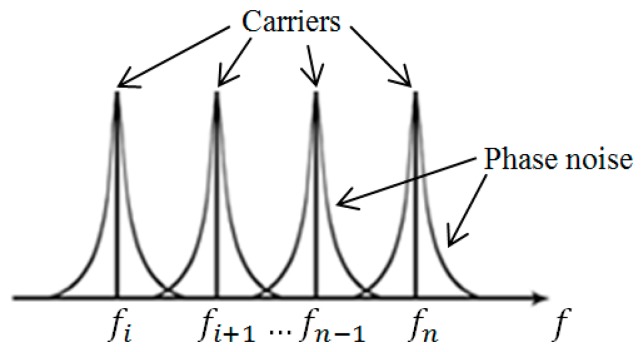
OFDM signal of multiple carriers, each carrier frequency has been distorted by the phase noise.

Phase noise is introduced when there is a small deviation of phase of a RF signal in oscillators and such deviation is commonly found in wireless communication systems. In LTE communication from BS to MH, signal transmission is in the form of multiple carriers of OFDM system. The corresponding phase noise [13] associated with multiple carriers in OFDM are shown in Figure 1. From this figure, it is seen that phase noise in neighboring channels may influence one another and is additive in nature.

Phase noise is a spectral density of phase deviation and usually represented as L(*f*) and expressed in dBc/Hz. The phase noise in a noisy oscillator can be approximated by a Leeson function [14]. In a multi-carrier system, an aggregate effect of phase noise needs to be considered. Assume that an OFDM symbol has n carriers, where f*_i_* is the carrier frequency, Δ*f* is the offset frequency, F is the noise factor, *P_s_* is the oscillator signal power level, *Q* is the quality factor, k is the Boltzmann constant and T is the temperature in Kelvin. By observing the waveform and by intuition, the individual phase noise is represented in Equation (2) where m is to be determined and A is a constant to be characterized. An experiment was performed on the effective offset that should be included at 2 GHz. In the design, it was found from experiment that *A*/*QP_s_* was typically in the range ∼800–1600. In our design, *A*/*QP_s_* = 1200 and F = 10 were chosen. From measurement, it was concluded that an offset frequency Δ*f* of about ±0.00002% at 2 GHz accounted for most of the phase noise effect and that m had the best fitting in the range between 1.5 and 2.5:

(2) $$\text{L}(\Delta f)(\text{dBc}/Hz)=(\text{AkTF}/QP_{s}){\left(f_{i}/\left|\Delta f\right|\right)}^{m}$$

Since there are multiple of carriers in LTE, the total phase noise in the system becomes:

(3) $$\int \text{L}(\Delta f)d\Delta f=\sum_{i=1}^{n}\int_{-0.00002\%f_{i}}^{+0.00002\%f_{i}}(\text{AkTF}/QP_{s}){\left(f_{i}/\left|\Delta f\right|\right)}^{m}d\Delta f$$

By incorporating the phase noise for multiple carriers in LTE, a new noise model has been developed and is shown in Equation (3). The effectiveness of the new model will be investigated in the next section.

## 3. Experimental Results

In high traffics areas, noise is a particularly prominent factor that may affect the accuracy of localization. The localization method in [9] is implemented by incorporating the developed noise model in Equation (3). The outdoor localization model is then modelled as:

(4) $$10\log_{10}\;\left(\frac{P_{t}}{P_{r}}\right)\;\left(\text{DBm}\right)=P_{L}+10\alpha \log_{10}d_{k}+w_{o}+\int \text{L}(\Delta f)d\Delta f$$

**Figure 2.**
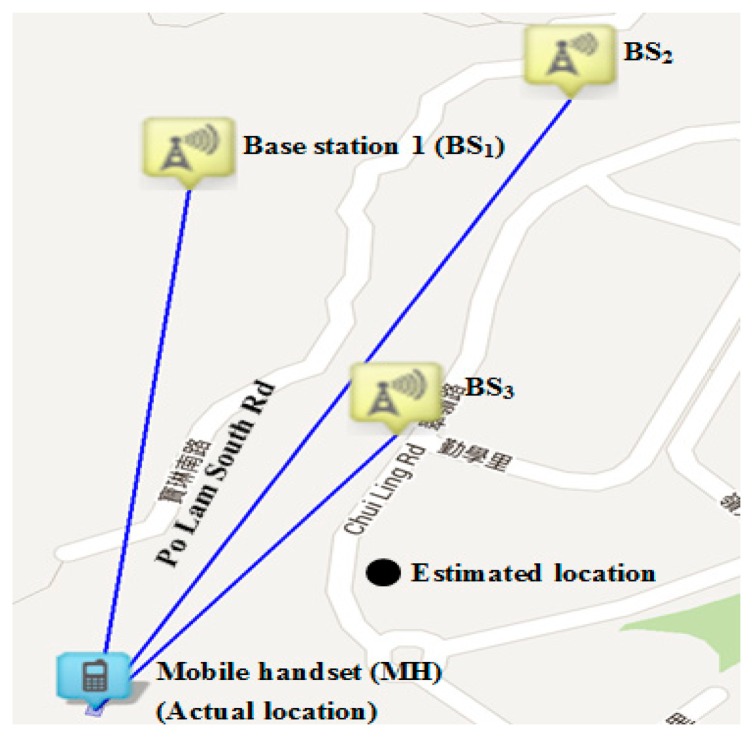
Localization experiment using new noise model (3) in a residential area in Hong Kong with variance ∼10, percentage improvement = 10%.

The localization model developed in Equation (4) (referred as the new scheme) is then used to evaluate the localization accuracy and comparison with the existing widely adopted localization scheme (1) (referred to as reference scheme) will be performed. The experiment was carried out in a noisy and yet busy area in the city of Hong Kong where there were more base stations than a typical remote area. During the experiment, a stationary MH was anchored into the actual location to intercept the RSS from neighboring BSs. By substituting the coordinates of all BSs and RSS values into Equation (4), an estimated location as shown in Figure 2 is obtained. The variance of random Gaussian noise typically varies according to condition of the environment under investigation. In our experiment, it was measured that the variance for random Gaussian noise was typically between 5 dB and 13 dB.

**Figure 3.**
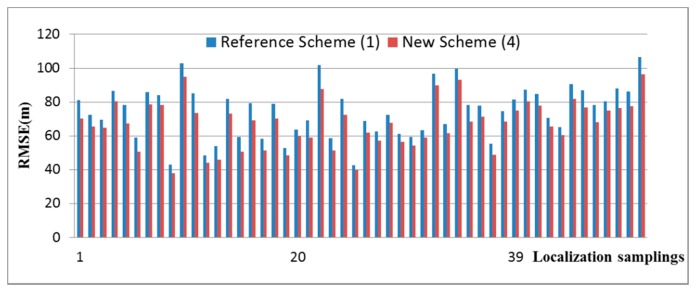
Comparison of reference scheme (1) and new scheme (4).

**Figure 4.**
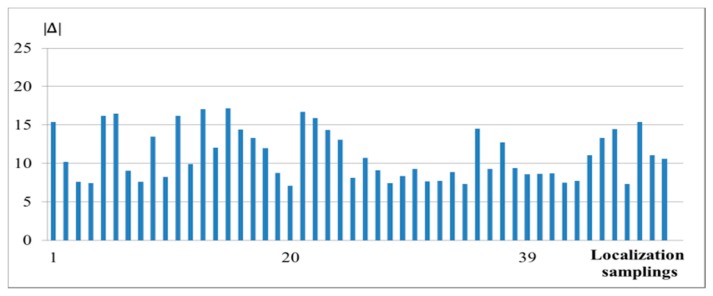
Percentage improvement of accuracy of the new scheme (4) over the reference scheme (1), |Δ|, based on 50 localization samplings in residential area in Hong Kong.

Fifty (50) RSSs data were captured by a MH and used for the RSS localization. Measurement data using the reference scheme are recorded and compared with the new scheme in Figure 3. The percentage improvement of accuracy of the new scheme (4) over the reference scheme (1), |Δ|, based on 50 localization samplings is shown in Figure 4. It is seen that the accuracy has been improved by about 10% on average.

## 4. Conclusions

An accurate localization model using Long Term Evolution (LTE) has been developed by incorporating a high accuracy noise model. The developed noise model incorporates the multiple carrier effect of the phase noise in LTE. Basic parameters for phase noise of carriers at 2 GHz have been characterized. The new localization model, based on the developed noise model, has been applied to RSS localization of LTE in a busy residential area in Hong Kong. The new localization method achieves on average an accuracy improvement of about 10%.
